# Supplementary material for: Arrayed CRISPRi and quantitative imaging describe the morphotypic landscape of essential mycobacterial genes
Source: eLife. 2020 Nov 6;9:e60083. doi: 10.7554/eLife.60083 (PMC7647400; doi:10.7554/eLife.60083)
Supplement: Supplementary file 4. [file elife-60083-supp4.docx]

**SUPPLEMENTARY METHODS**

**Sequencing PCR Reaction**

Sequencing Primers (5’ to 3’):

Forward: CTGGTCCACCTACAACAAAG

Reverse: CCCTGATTCTGTGGATAACC

|  |  |  |
| --- | --- | --- |
| **STEP** | **TEMP** | **TIME** |
| Initial Denaturation | 94°C | 30 seconds |
| 30 Cycles | 94°C 49°C 68°C | 15-30 seconds 15-60 seconds 1 minute/kb |
| Final Extension | 68°C | 5 minutes |
| Hold | 4-10°C |  |

**Golden Gate PCR Reaction**

Golden Gate Primers (5’ to 3’)::

Forward: ACTTCGGCTCTTCG*GGA*TCTGACCAGGGAAAATAGC

Reverse: ACTTCGGCTCTTCGCTGAAAATAAAAAAGGGGACCTCTAG

|  |  |  |
| --- | --- | --- |
| **STEP** | **TEMP** | **TIME** |
| Initial Denaturation | 98°C | 2 minutes |
| 30 Cycles | 98°C 50°C 72°C | 10 seconds 30 seconds 30 seconds |
| Final Extension | 72°C | 3 minutes |
| Hold | 4 |  |
